# (1*E*,4*E*)-1,5-Bis(4-methyl­phen­yl)penta-1,4-dien-3-one

**DOI:** 10.1107/S1600536808020084

**Published:** 2008-07-05

**Authors:** Muhammad Nadeem Arshad, Muhammad Nawaz Tahir, Muhammad Nadeem Asghar, Islam Ullah Khan, Muhammad Ashfaq

**Affiliations:** a Government College University, Department of Chemistry, Lahore, Pakistan; b University of Sargodha, Department of Physics, Sargodha, Pakistan

## Abstract

The title compound, C_19_H_18_O, crystallizes in a non-centrosymmetric space group although the mol­ecule has no chiral centre. The dihedral angle between the aromatic rings is 20.43 (13)°. The structure is stabilized by two intra­molecular hydrogen bonds, and by four π–π and three C—H⋯π inter­actions between the aromatic rings. The perpendicular distances between the centroids of the rings involved in the π–π inter­actions have values of 1.996, 2.061, 2.181 and 2.189 Å.

## Related literature

For related literature, see: Butcher *et al.* (2006 ▶); Conard & Dolliver (1943 ▶); Harrison *et al.* (2006 ▶).
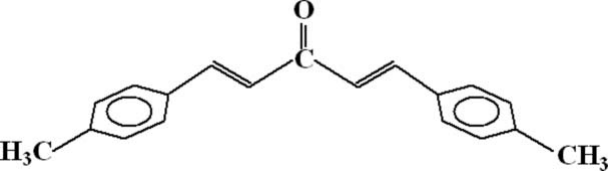


## Experimental

### 

#### Crystal data

C_19_H_18_O
*M* *_r_* = 262.33
Monoclinic, 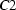

*a* = 19.937 (2) Å
*b* = 5.8637 (5) Å
*c* = 14.9207 (14) Å
β = 121.001 (3)°
*V* = 1495.1 (2) Å^3^
*Z* = 4
Mo *K*α radiation radiation
μ = 0.07 mm^−1^
*T* = 296 (2) K
0.25 × 0.20 × 0.15 mm

#### Data collection

Bruker Kappa APEXII CCD diffractometer
Absorption correction: multi-scan (*SADABS*; Bruker, 2005 ▶) *T*_min_ = 0.980, *T*_max_ = 0.988
9611 measured reflections
2288 independent reflections
1800 reflections with *I* > 2σ(*I*)
*R*_int_ = 0.024

#### Refinement

*R*[*F* ^2^ > 2σ(*F* ^2^)] = 0.048
*wR*(*F* ^2^) = 0.137
*S* = 1.04
2288 reflections
183 parameters
1 restraint
H-atom parameters constrained
Δρ_max_ = 0.22 e Å^−3^
Δρ_min_ = −0.21 e Å^−3^

### 

Data collection: *APEX2* (Bruker, 2007 ▶); cell refinement: *APEX2*; data reduction: *SAINT* (Bruker, 2007 ▶); program(s) used to solve structure: *SHELXS97* (Sheldrick, 2008 ▶); program(s) used to refine structure: *SHELXL97* (Sheldrick, 2008 ▶); molecular graphics: *ORTEP-3 for Windows* (Farrugia, 1997 ▶) and *PLATON* (Spek, 2003 ▶); software used to prepare material for publication: *WinGX* (Farrugia, 1999 ▶) and *PLATON*.

## Supplementary Material

Crystal structure: contains datablocks global, I. DOI: 10.1107/S1600536808020084/bq2087sup1.cif

Structure factors: contains datablocks I. DOI: 10.1107/S1600536808020084/bq2087Isup2.hkl

Additional supplementary materials:  crystallographic information; 3D view; checkCIF report

## Figures and Tables

**Table 1:** Hydrogen-bond geometry (Å, °) *CgA* and *CgB* are the centroids of the C4–C9 and C13–C18 rings, respectively.

*D*—H⋯*A*	*D*—H	H⋯*A*	*D*⋯*A*	*D*—H⋯*A*
C3—H3⋯O1	0.93	2.49	2.817 (4)	101
C12—H12⋯O1	0.93	2.48	2.819 (3)	102
C5—H5⋯*CgA*^i^	0.93	2.82	3.523 (3)	133
C9—H9⋯*CgB*^ii^	0.93	2.89	3.604 (3)	134
C18—H18⋯*CgB*^iii^	0.93	2.95	3.621 (3)	131

Symmetry codes: (i) 
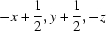
; (ii) 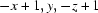; (iii) 
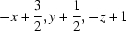
.

**Fig. 1.**
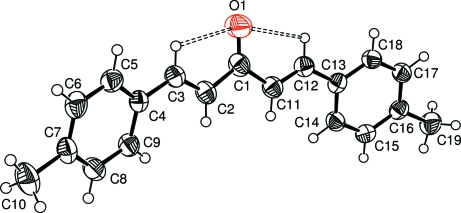
ORTEP drawing of the title compound, with the atom numbering scheme. The thermal ellipsoids are drawn at the 50% probability level. H-atoms are shown by small circles of arbitrary radii. The intramolecular H-bonding is shown by dotted lines.

**Fig. 2.**
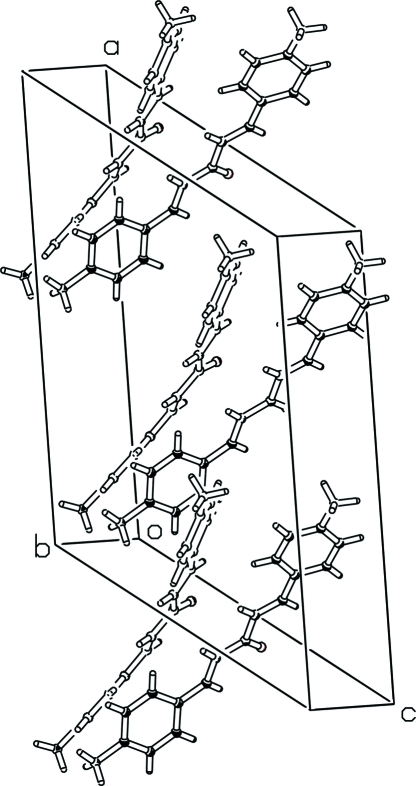
The packing figure (PLATON: Spek, 2003) which shows the stacking of molecules in a unit cell.

## supplementary crystallographic information

### Comment

The chalcones are being synthesized since long for various applications. The title compound (I) differs from 1,5-Bis(4-methoxyphenyl)penta-1,4- dien-3-one (II) (Harrison *et al.*, 2006) and 1,5-Bis(4-chlorophenyl)penta-1,4- dien-3-one (III) (Butcher *et al.*, 2006) due the attachement of methyl group at *para* position instead of methoxy or chloro moieties. The authors have reported their nonlinear optical (NLO) activity as the compounds crystallize in non-centrosymmetric space groups. The title compound have a monoclinic crystal system and a non-centrosymmetric space group C2. Therefore, it is assumed that this compound will also have nonlinear optical activity.

We compare the bond distances and bond angles realised in (I) with the corresponding values observed in (II) and (III). The central portion of (I) shows double and single bonds originating from O1-atom and five-C-atom behave like a backbone. In (I), the bond distance O1==C1 is 1.211 (4) Å, greater than 1.170 (12) Å (III) but shorter than 1.230 (6) Å as in (II). The bond distances C1—C2 = 1.480 (3) Å, C2==C3 = 1.329 (3) Å, C3—C4 = 1.465 (3) Å are observed in (I), in (II) and (III) the corresponding values are [1.476 (4), 1.318 (5), 1.475 (4) Å] and [1.579 (10), 1.239 (7), 1.502 (7) Å], respectively. The range of bond angles for backbone C-atoms in (I) is 116.0 (2)°-127.8 (2)°, whereas in (II) and (III), the range is 123.8 (5)°- 126.4 (4)° and 103.1 (9)°-128.5 (4)°, respectively. The dihedral angle between the aromatic rings A (C4—C9) and B (C13—C18) is 20.27 (13)°, which is less than 56.92 (9)° and 53.4 (5) as reported in (II) and (III), respectively. The title compound is stabilized due to two intramolecular H-bonding (Fig 1) and three C—H···π interactions as given in Table 1. There exist also π–π-interactions between the aromatic rings. The perpendicular distance between the centroids CgA and CgB [(CgA···CgA^iv^), symmetry code: iv = -*x* + 1/2, *y* - 1/2, -*z*], [(CgA···CgB^v^), symmetry code: v = -*x* + 1, *y* + 1, -*z* + 1], [(CgB···CgA^ii^), symmetry code: ii = -*x* + 1, *y*, -*z* + 1] and [(CgB···CgB^vi^), symmetry code: vi = -*x* + 3/2, *y* - 1/2, -*z* + 1] have values of 1.996, 2.181, 2.061 and 2.189 Å, respectively.

### Experimental

The title compound (I) was synthesized using the method of Conard & Dolliver, 1943. Sodium hydroxide (0.8 g, 0.0208 mmol) was dissolved in distilled water (10 ml) and ethanol (8 ml) in a round bottom flask. The solution was cooled to room temperature. Half of the mixture of *p*-tolualdehide (1 g, 0.00833 mmol) and acetone (0.24 g, 0.00417 mmol) added to the above solution and stirred at room temperature for 15 minute then the remaining mixture was added and stirred for 2 h under the same conditions. Yellow precipitate obtained was filtered and washed with cold water. The washed precipitate was crystallized in aceton under slow evaporation.

### Refinement

H-atoms were positioned geometrically, with C—H = 0.93 and 0.96 Å for aromatic and methyl H, and constrained to ride on their parent atoms, with *U*_iso_(H) = *xU*_eq_(C), where *x* = 1.5 for methyl H, and *x* = 1.2 for other H atoms. Friedel pairs were averaged before the final refinement as the absolute configuration could not be determined unambiguously.

### Crystal data

**Table:**

C_19_H_18_O	*F*_000_ = 560
*M**_r_* = 262.33	*D*_x_ = 1.165 Mg m^−^^3^
Monoclinic, *C*2	Mo *K*α radiation radiation λ = 0.71073 Å
Hall symbol: C 2y	Cell parameters from 3173 reflections
*a* = 19.937 (2) Å	θ = 2.1–29.6º
*b* = 5.8637 (5) Å	µ = 0.07 mm^−^^1^
*c* = 14.9207 (14) Å	*T* = 296 (2) K
β = 121.001 (3)º	Prism, yellow
*V* = 1495.1 (2) Å^3^	0.25 × 0.20 × 0.15 mm
*Z* = 4	

### Data collection

**Table:**

Bruker Kappa APEXII CCD diffractometer	2288 independent reflections
Radiation source: fine-focus sealed tube	1800 reflections with *I* > 2σ(*I*)
Monochromator: graphite	*R*_int_ = 0.024
Detector resolution: 7.40 pixels mm^-1^	θ_max_ = 29.6º
*T* = 296(2) K	θ_min_ = 2.1º
ω scans	*h* = −26→27
Absorption correction: multi-scan(SADABS; Bruker, 2005)	*k* = −8→4
*T*_min_ = 0.980, *T*_max_ = 0.988	*l* = −20→18
9611 measured reflections	

### Refinement

**Table:**

Refinement on *F*^2^	Secondary atom site location: difference Fourier map
Least-squares matrix: full	Hydrogen site location: inferred from neighbouring sites
*R*[*F*^2^ > 2σ(*F*^2^)] = 0.048	H-atom parameters constrained
*wR*(*F*^2^) = 0.137	*w* = 1/[σ^2^(*F*_o_^2^) + (0.0717*P*)^2^ + 0.4143*P*] where *P* = (*F*_o_^2^ + 2*F*_c_^2^)/3
*S* = 1.04	(Δ/σ)_max_ < 0.001
2288 reflections	Δρ_max_ = 0.22 e Å^−^^3^
183 parameters	Δρ_min_ = −0.21 e Å^−^^3^
1 restraint	Extinction correction: none
Primary atom site location: structure-invariant direct methods	Absolute structure: Flack (1983), 726 Friedel pairs

### Special details

**Table:**

Geometry. All e.s.d.'s (except the e.s.d. in the dihedral angle between two l.s. planes) are estimated using the full covariance matrix. The cell e.s.d.'s are taken into account individually in the estimation of e.s.d.'s in distances, angles and torsion angles; correlations between e.s.d.'s in cell parameters are only used when they are defined by crystal symmetry. An approximate (isotropic) treatment of cell e.s.d.'s is used for estimating e.s.d.'s involving l.s. planes.
Refinement. Refinement of *F*^2^ against ALL reflections. The weighted *R*-factor *wR* and goodness of fit *S* are based on *F*^2^, conventional *R*-factors *R* are based on *F*, with *F* set to zero for negative *F*^2^. The threshold expression of *F*^2^ > σ(*F*^2^) is used only for calculating *R*-factors(gt) *etc*. and is not relevant to the choice of reflections for refinement. *R*-factors based on *F*^2^ are statistically about twice as large as those based on *F*, and *R*- factors based on ALL data will be even larger.

### Fractional atomic coordinates and isotropic or equivalent isotropic displacement parameters (Å^2^)

**Table:**

	*x*	*y*	*z*	*U*_iso_*/*U*_eq_	
O1	0.49937 (12)	1.0189 (4)	0.63720 (18)	0.0745 (7)	
C1	0.51762 (14)	0.8202 (5)	0.6423 (2)	0.0495 (6)	
C2	0.59839 (13)	0.7416 (5)	0.71598 (18)	0.0470 (6)	
H2	0.6115	0.5906	0.7132	0.056*	
C3	0.65267 (13)	0.8809 (4)	0.78604 (17)	0.0429 (5)	
H3	0.6374	1.0308	0.7861	0.051*	
C4	0.73402 (12)	0.8232 (4)	0.86323 (17)	0.0383 (5)	
C5	0.77901 (14)	0.9756 (4)	0.94391 (19)	0.0469 (5)	
H5	0.7576	1.1150	0.9460	0.056*	
C6	0.85484 (15)	0.9234 (5)	1.0209 (2)	0.0520 (6)	
H6	0.8835	1.0278	1.0741	0.062*	
C7	0.88884 (13)	0.7179 (5)	1.02006 (18)	0.0482 (6)	
C8	0.84507 (14)	0.5688 (5)	0.9386 (2)	0.0523 (6)	
H8	0.8672	0.4316	0.9355	0.063*	
C9	0.76890 (13)	0.6194 (4)	0.86135 (18)	0.0459 (6)	
H9	0.7407	0.5157	0.8076	0.055*	
C10	0.97104 (15)	0.6567 (8)	1.1064 (2)	0.0751 (10)	
H10A	0.9802	0.7163	1.1717	0.113*	
H10B	1.0082	0.7211	1.0910	0.113*	
H10C	0.9767	0.4938	1.1112	0.113*	
C11	0.46029 (14)	0.6396 (4)	0.57787 (19)	0.0474 (6)	
H11	0.4775	0.4907	0.5810	0.057*	
C12	0.38539 (13)	0.6886 (5)	0.51634 (17)	0.0439 (5)	
H12	0.3713	0.8398	0.5164	0.053*	
C13	0.32183 (13)	0.5337 (4)	0.44805 (17)	0.0405 (5)	
C14	0.33464 (13)	0.3184 (4)	0.41838 (19)	0.0447 (5)	
H14	0.3856	0.2673	0.4444	0.054*	
C15	0.27265 (14)	0.1824 (4)	0.35119 (18)	0.0444 (5)	
H15	0.2822	0.0410	0.3318	0.053*	
C16	0.19537 (14)	0.2537 (4)	0.31160 (18)	0.0432 (5)	
C17	0.18269 (14)	0.4658 (4)	0.34177 (19)	0.0455 (5)	
H17	0.1318	0.5159	0.3167	0.055*	
C18	0.24460 (13)	0.6022 (4)	0.40822 (18)	0.0438 (5)	
H18	0.2348	0.7438	0.4271	0.053*	
C19	0.12788 (15)	0.1027 (5)	0.2395 (2)	0.0596 (7)	
H19A	0.1447	−0.0047	0.2065	0.089*	
H19B	0.0862	0.1944	0.1871	0.089*	
H19C	0.1097	0.0220	0.2790	0.089*	

### Atomic displacement parameters (Å^2^)

**Table:**

	*U*^11^	*U*^22^	*U*^33^	*U*^12^	*U*^13^	*U*^23^
O1	0.0515 (11)	0.0599 (12)	0.0763 (14)	0.0052 (10)	0.0073 (10)	−0.0046 (11)
C1	0.0385 (12)	0.0582 (15)	0.0410 (13)	0.0011 (11)	0.0128 (10)	−0.0008 (12)
C2	0.0365 (11)	0.0545 (14)	0.0420 (12)	0.0027 (11)	0.0146 (10)	0.0004 (11)
C3	0.0392 (11)	0.0471 (12)	0.0406 (12)	0.0013 (11)	0.0194 (10)	0.0008 (10)
C4	0.0339 (10)	0.0444 (11)	0.0356 (11)	−0.0026 (9)	0.0171 (9)	0.0000 (10)
C5	0.0474 (13)	0.0427 (11)	0.0500 (13)	−0.0041 (10)	0.0247 (11)	−0.0086 (11)
C6	0.0436 (13)	0.0587 (15)	0.0446 (13)	−0.0127 (12)	0.0162 (11)	−0.0164 (12)
C7	0.0342 (11)	0.0621 (15)	0.0434 (13)	−0.0031 (11)	0.0164 (10)	0.0021 (13)
C8	0.0422 (12)	0.0522 (14)	0.0591 (15)	0.0055 (11)	0.0236 (11)	−0.0037 (13)
C9	0.0398 (12)	0.0466 (12)	0.0456 (13)	−0.0060 (10)	0.0179 (10)	−0.0144 (11)
C10	0.0380 (13)	0.098 (3)	0.0664 (18)	0.0033 (17)	0.0103 (13)	0.0052 (19)
C11	0.0432 (12)	0.0502 (13)	0.0443 (13)	0.0016 (11)	0.0193 (10)	0.0002 (11)
C12	0.0431 (12)	0.0469 (11)	0.0406 (12)	0.0004 (11)	0.0208 (10)	0.0016 (10)
C13	0.0415 (11)	0.0430 (11)	0.0341 (11)	−0.0038 (10)	0.0173 (9)	0.0034 (10)
C14	0.0403 (11)	0.0485 (12)	0.0451 (12)	0.0046 (10)	0.0219 (10)	0.0066 (11)
C15	0.0518 (13)	0.0388 (10)	0.0449 (12)	−0.0001 (10)	0.0265 (11)	0.0004 (10)
C16	0.0461 (12)	0.0426 (11)	0.0384 (11)	−0.0039 (10)	0.0201 (10)	0.0035 (9)
C17	0.0368 (11)	0.0469 (12)	0.0483 (13)	0.0041 (10)	0.0187 (10)	0.0076 (11)
C18	0.0445 (12)	0.0418 (11)	0.0459 (13)	0.0042 (10)	0.0238 (10)	0.0041 (10)
C19	0.0507 (14)	0.0595 (16)	0.0560 (15)	−0.0103 (13)	0.0185 (12)	−0.0065 (14)

### Geometric parameters (Å, °)

**Table:**

O1—C1	1.211 (4)	C10—H10C	0.9600
C1—C2	1.480 (3)	C11—C12	1.320 (3)
C1—C11	1.490 (4)	C11—H11	0.9300
C2—C3	1.329 (3)	C12—C13	1.463 (3)
C2—H2	0.9300	C12—H12	0.9300
C3—C4	1.465 (3)	C13—C18	1.394 (3)
C3—H3	0.9300	C13—C14	1.404 (3)
C4—C9	1.390 (3)	C14—C15	1.375 (3)
C4—C5	1.393 (3)	C14—H14	0.9300
C5—C6	1.382 (4)	C15—C16	1.400 (3)
C5—H5	0.9300	C15—H15	0.9300
C6—C7	1.386 (4)	C16—C17	1.390 (3)
C6—H6	0.9300	C16—C19	1.503 (3)
C7—C8	1.383 (4)	C17—C18	1.373 (3)
C7—C10	1.516 (3)	C17—H17	0.9300
C8—C9	1.385 (3)	C18—H18	0.9300
C8—H8	0.9300	C19—H19A	0.9600
C9—H9	0.9300	C19—H19B	0.9600
C10—H10A	0.9600	C19—H19C	0.9600
C10—H10B	0.9600		
			
O1—C1—C2	121.5 (3)	H10B—C10—H10C	109.5
O1—C1—C11	122.5 (2)	C12—C11—C1	120.7 (2)
C2—C1—C11	116.0 (2)	C12—C11—H11	119.6
C3—C2—C1	121.8 (2)	C1—C11—H11	119.6
C3—C2—H2	119.1	C11—C12—C13	127.8 (2)
C1—C2—H2	119.1	C11—C12—H12	116.1
C2—C3—C4	126.8 (2)	C13—C12—H12	116.1
C2—C3—H3	116.6	C18—C13—C14	117.7 (2)
C4—C3—H3	116.6	C18—C13—C12	119.2 (2)
C9—C4—C5	117.6 (2)	C14—C13—C12	123.0 (2)
C9—C4—C3	123.1 (2)	C15—C14—C13	120.7 (2)
C5—C4—C3	119.4 (2)	C15—C14—H14	119.7
C6—C5—C4	121.2 (2)	C13—C14—H14	119.7
C6—C5—H5	119.4	C14—C15—C16	121.0 (2)
C4—C5—H5	119.4	C14—C15—H15	119.5
C5—C6—C7	121.1 (2)	C16—C15—H15	119.5
C5—C6—H6	119.5	C17—C16—C15	118.3 (2)
C7—C6—H6	119.5	C17—C16—C19	120.9 (2)
C8—C7—C6	117.9 (2)	C15—C16—C19	120.7 (2)
C8—C7—C10	120.9 (3)	C18—C17—C16	120.6 (2)
C6—C7—C10	121.2 (3)	C18—C17—H17	119.7
C7—C8—C9	121.4 (3)	C16—C17—H17	119.7
C7—C8—H8	119.3	C17—C18—C13	121.7 (2)
C9—C8—H8	119.3	C17—C18—H18	119.2
C8—C9—C4	120.9 (2)	C13—C18—H18	119.2
C8—C9—H9	119.6	C16—C19—H19A	109.5
C4—C9—H9	119.6	C16—C19—H19B	109.5
C7—C10—H10A	109.5	H19A—C19—H19B	109.5
C7—C10—H10B	109.5	C16—C19—H19C	109.5
H10A—C10—H10B	109.5	H19A—C19—H19C	109.5
C7—C10—H10C	109.5	H19B—C19—H19C	109.5
H10A—C10—H10C	109.5		
			
O1—C1—C2—C3	6.0 (4)	O1—C1—C11—C12	−4.0 (4)
C11—C1—C2—C3	−172.2 (2)	C2—C1—C11—C12	174.1 (2)
C1—C2—C3—C4	179.6 (2)	C1—C11—C12—C13	179.6 (2)
C2—C3—C4—C9	11.2 (4)	C11—C12—C13—C18	164.8 (2)
C2—C3—C4—C5	−167.6 (2)	C11—C12—C13—C14	−16.6 (4)
C9—C4—C5—C6	−1.7 (4)	C18—C13—C14—C15	0.8 (3)
C3—C4—C5—C6	177.1 (2)	C12—C13—C14—C15	−177.9 (2)
C4—C5—C6—C7	0.4 (4)	C13—C14—C15—C16	−0.7 (3)
C5—C6—C7—C8	1.3 (4)	C14—C15—C16—C17	0.1 (3)
C5—C6—C7—C10	−177.9 (3)	C14—C15—C16—C19	−179.0 (2)
C6—C7—C8—C9	−1.6 (4)	C15—C16—C17—C18	0.4 (3)
C10—C7—C8—C9	177.6 (3)	C19—C16—C17—C18	179.5 (2)
C7—C8—C9—C4	0.2 (4)	C16—C17—C18—C13	−0.3 (3)
C5—C4—C9—C8	1.5 (4)	C14—C13—C18—C17	−0.3 (3)
C3—C4—C9—C8	−177.4 (2)	C12—C13—C18—C17	178.4 (2)

### Hydrogen-bond geometry (Å, °)

**Table:**

*D*—H···*A*	*D*—H	H···*A*	*D*···*A*	*D*—H···*A*
C3—H3···O1	0.93	2.49	2.817 (4)	101
C12—H12···O1	0.93	2.48	2.819 (3)	102
C5—H5···CgA^i^	0.93	2.82	3.523 (3)	133
C9—H9···CgB^ii^	0.93	2.89	3.604 (3)	134
C18—H18···CgB^iii^	0.93	2.95	3.621 (3)	131

Symmetry codes: (i) −*x*+1/2, *y*+1/2, −*z*; (ii) −*x*+1, *y*, −*z*+1; (iii) −*x*+3/2, *y*+1/2, −*z*+1.
